# Tick-borne pathogens in *Ixodes ricinus* ticks collected from migratory birds in southern Norway

**DOI:** 10.1371/journal.pone.0230579

**Published:** 2020-04-09

**Authors:** Benedikte N. Pedersen, Andrew Jenkins, Vivian Kjelland

**Affiliations:** 1 Department of Natural Science and Environmental Health, University of South-Eastern Norway, Gullbringvegen, Norway; 2 Department of Natural Sciences, Faculty of Engineering and Science, University of Agder, Kristiansand, Norway; 3 Sørlandet Hospital Health Enterprise, Research Unit, Kristiansand, Norway; University of Kentucky College of Medicine, UNITED STATES

## Abstract

Birds are important hosts for the first life stages of the *Ixodes ricinus* tick and they can transport their parasites over long distances. The aim of this study was to investigate the prevalence of *Borrelia burgdorferi* sensu lato, *Anaplasma phagocytophilum*, *Neoehrlichia mikurensis* and *Rickettsia helvetica* in ticks collected from migratory birds in Norway. A total of 815 *Ixodes ricinus* ticks from 216 birds trapped at Lista Bird Observatory in southern Norway during spring and autumn migration in 2008 were analysed by real-time PCR. *B*. *burgdorferi* s. l. was the most prevalent pathogen, detected in 6.1% of the ticks. The prevalence of *N*. *mikurensis*, *A*. *phagocytophilum* and *R*. *helvetica* was 1.2%, 0.9% and 0.4% respectively. In addition, one sample (0.1%) was positive for *B*. *miyamotoi*. In total, 8.2% of the ticks were infected with at least one pathogen. Co-infection with *B*. *burgdorferi* s. l. and *N*. *mikurensis* or *A*. *phagocytophilum* was found in 6.0% of the infected ticks. Our results show that all the known major tick-borne bacterial pathogens in Norway are subject to transport by migratory birds, potentially allowing spread to new areas. Our study showed a surprisingly high number of samples with PCR inhibition (57%). These samples had been extracted using standard methodology (phenol-chloroform extraction). This illustrates the need for inhibition controls to determine true prevalence rates.

## Introduction

The tick *Ixodes ricinus* is the main vector of several pathogens important for human and animal health in Europe. Passerine birds are significant hosts for the first life stages of *I*. *ricinus* and migratory birds may transport parasites across continents along the migration routes [1, 2]. Several studies from Europe have investigated tick-borne pathogens transported by migrating birds and *Borrelia burgdorferi* sensu lato (s. l.), *Rickettsia helvetica*, *Anaplasma phagocytophilum* and *Neoehrlichia mikurensis* are some of the pathogens detected in *I*. *ricinus* ticks feeding on birds [3–6].

One of the most important tick-borne pathogens and the causative agent of Lyme disease is *B*. *burgdorferi* s. l. The prevalence of the spirochete in *I*. *ricinus* feeding on birds is normally lower than 30% [6–9], however, infection rates exceeding this has been reported occasionally [3, 5]. *B*. *garinii* and *B*. *valaisiana* are the most prevalent of the *Borrelia* species detected in ticks feeding on birds [3, 6], and the blackbird (*Turdus merula*) and song thrush (*T*. *philomeus*) are assumed to be reservoir hosts for these species [10, 11].

One of the most prevalent tick-borne pathogens in questing *I*. *ricinus* in Norway is *N*. *mikurensis* [12–14], although, so far only one case of neoehrlichiosis has been reported in this country [15]. *N*. *mikurensis* is detected in *I*. *ricinus* collected from birds, and the prevalence is typically below 5% [3, 5, 16], but *N*. *mikurensis* has so far not been detected in blood samples from passerine birds [17]. Furthermore, a study investigating the great tit (*Parus major*) failed to find evidence of amplification and transmission [18].

Tick-borne fever, or anaplasmosis, in ruminants is caused by *A*. *phagocytophilum*. Tick-borne fever is common in sheep in Norway, and the disease is found in herds grazing on tick infested pastures [19, 20]. The prevalence of the bacterium in *I*. *ricinus* ticks feeding on birds is typically below 5% [3, 5–7]. *A*. *phagocytophilum* has also been detected in blood samples from passerine birds, suggesting they are capable of transmitting the bacterium to ticks [17, 21]. However, the importance of birds for the infectious cycle of *A*. *phagocytophilum* is unclear.

The only bacterium in the spotted fever group *Rickettsiae* detected in Norway so far is *R*. *helvetica* [22], Kjelland, Myre, Pedersen, Kloster, & Jenkins, Submitted manuscript). Although the bacterium is present in questing ticks in Norway no cases of rickettsiosis have been reported so far. Prevalences ranging from about 10% to 20% of *R*. *helvetica* in *I*. *ricinus* collected from birds are reported in Europe [3, 5–7]. It has been demonstrated that the great tit (*Parus major*) facilitates transmission of *R*. *helvetica* to *I*. *ricinus* [18]. The bacterium has been detected in blood samples from other passerine birds, indicating a potential reservoir capacity [17]. Although further research is necessary to determine to which degree birds contribute to the infection of ticks, they are important for the epidemiological distribution of *R*. *helvetica*.

Previously in Norway, three studies have investigated tick-borne pathogens transported by migrating birds, focusing on *B*. *burgdorferi* s. l. or *A*. *phagocytophilum* [4, 8, 23]. Studies of more recently identified pathogens in this region are absent. Kjelland et al. [4] have previously studied the prevalence of *B*. *burgdorferi* s. l. in ticks collected from migratory birds in southern Norway. The present work is an extension of the latter study, aimed at bird contribution to dispersal of tick-borne bacteria, by further investigating the same sample material for *A*. *phagocytophilum*, *N*. *mikurensis* and *R*. *helvetica*, and determining the prevalence of co-infection.

## Materials and methods

Bird trapping, collection of ticks and preparation of samples have been described previously by Kjelland et al. [4]. Briefly, 6538 birds were trapped at Lista Bird Observatory in southern Norway during spring (April to June) and autumn (July to November) migration in 2008. The birds were trapped for ringing and an ethics approval was not needed. Birds were examined and ticks were removed from both migratory and resident species. The sample material included a total of 815 ticks, whereof 201 ticks were collected from 64 birds during spring migration and 614 ticks were collected from 152 birds during autumn migration. Only larval and nymphal *I*. *ricinus* were found. DNA was extracted by the phenol-chloroform method and analysed for *B*. *burgdorferi* s. l.

In the present study, the ticks were investigated for *N*. *mikurensis*, *R*. *helvetica*, and *A*. *phagocytophilum*. Real-time PCR methods for detection of the pathogens were performed as described previously [12, 24, Kjelland, Myre, Pedersen, Kloster, & Jenkins, Submitted manuscript]. Synthetic plasmids were used as positive controls for *A*. *phagocytophilum* and *N*. *mikurensis* real-time PCR; a synthetic plasmid or a known positive sample was used as control for *R*. *helvetica* in real-time PCR and pyrosequencing. Nuclease free water was used as negative control and included in all analyses. All primers and probes used in this study are listed in Table 1.

**Table 1:** Sequences for primers and probes used in present study.

Primers and probes	Sequence 5’ to 3’	Reference
*N*. *mikurensis* forward primer (Neo2F)	GCAAATGGAGATAAAAACATAGGTAGTAAA	[12]
*N*. *mikurensis* reverse primer (Neo2R)	CATACCGTCAGTTTTTTCAACTTCTAA	[12]
*N*. *mikurensis* probe (Neo2M)	FAM-TTACAGTTGAGGAAAGTAAGGGA(MGB)	[12]
*R*. *helvetica* forward primer (RhF)	CCGTTTAGGTTAATAGGCTTCGG	Kjelland, Myre, Pedersen, Kloster, & Jenkins, Submitted manuscript
*R*. *helvetica* forward primer, biotin labelled	CCGTTTAGGTTAATAGGCTTCGG	Kjelland, Myre, Pedersen, Kloster, & Jenkins, Submitted manuscript
*R*. *helvetica* reverse primer (RhR)	CCGAGTTCCTTTAATACTTCCTTACA	Kjelland, Myre, Pedersen, Kloster, & Jenkins, Submitted manuscript
*R*. *helvetica* probe (RhM)	VIC-CGATCCACGTGCCGCAGTACT(MGB)	Kjelland, Myre, Pedersen, Kloster, & Jenkins, Submitted manuscript
*A*. *phagocytophilum* forward primer (ApF)	TTTTGGGCGCTGAATACGAT	[24]
*A*. *phagocytophilum* reverse primer (ApR)	TCTCGAGGGAATGATCTAATAACGT	[24]
*A*. *phagocytophilum* probe (ApM)	FAM-TGCCTGAACAAGTTATG(MGB)	[24]
*B*. *burgdorferi* s. l. 16S rDNA forward primer (LBf)	GCTGTAAACGATGCACACTTGGT	[25]
*B*. *burgdorferi* s. l. reverse 16S rDNA primer (LBr)	GGCGGCACACTTAACACGTTAG	[25]
*B*. *burgdorferi* s. l. probe 16S rDNA (LBp)	FAM-TTCGGTACTAACTTTTAGTTAA(MGB)	[25]
*Borrelia* spp forward primer 1 (IGS F)	GTATGTTTAGTGAGGGGGGTG	[26]
*Borrelia* spp reverse primer 1 (IGS R)	GGATCATAGCTCAGGTGGTTAG	[26]
*Borrelia* spp nested forward primer 2(IGS Fn)	AGGGGGGTGAAGTCGTAACAAG	[26]
*Borrelia* spp nested reverse primer 2 (IGS Rn)	GTCTGATAAACCTGAGGTCGGA	[26]

Samples found negative for all pathogens were tested for inhibition. This was done by performing the *A*. *phagocytophilum* real-time PCR on the sample, using a master mix spiked with positive control, to ensure a positive result in non-inhibitory samples. All samples with Ct-values higher than the control itself were considered inhibitory. These samples were diluted 1:10, rechecked again for inhibition and thereafter reanalysed for all pathogens. The assays for detection of *R*. *helvetica* and *A*. *phagocytophilum* were at this point optimized for multiplexing. Every multiplex reaction was 15μl and included 7.5μl of 2x TaqMan Universal MasterMix (Applied Biosystems, Foster City, CA), 300nM of ApF and ApR, 250nM of ApM-FAM, 250nM of RhF and RhR, 300nM RhM-VIC, and 3μl of template DNA. The cycling parameters were as follows: 2 min at 50°C, 95°C for 10 min, then 45 cycles of 95°C for 15 s and 60°C for 1 min.

For confirmation, samples positive in the multiplex assay were reanalysed for both bacteria in individual reactions. Real-time PCR for detection of *R*. *helvetica* were performed with biotin labelled RhF and positive samples were subject to pyrosequencing on the Pyromark Q24 (Qiagen GmbH, Hilden, Germany) using Pyrogold reagents and primer RhR as previously described (Kjelland, Myre, Pedersen, Kloster, & Jenkins, Submitted manuscript). Inhibitory samples were also reanalysed for *B*. *burgdorferi* s. l. by real-time PCR and positive samples were confirmed by sequencing as previously described [4].

The chi-square test was performed using R v3.6.1 to determine significant difference in pathogen prevalence between spring and autumn migration.

## Results

In total, 17.1% (37/216) of the birds carried infected ticks, and 8.2% (67/815) of the ticks harboured tick-borne pathogen(s). The prevalence of pathogens was 10.9% (22/201) and 7.3% (45/614) during spring and autumn migration, respectively. This difference was not statistically significant (p = 0.1052). Because of the low number of positive samples, statistical testing for the individual pathogens was not attempted.

Initially, 771 of 815 samples were negative for all pathogens and of these 467 inhibited the PCR reaction, representing more than half (57%) of the material. Reanalysis of the inhibitory samples after dilution revealed additional pathogen-infected ticks. This increased the prevalence of *B*. *burgdorferi* s. l. from 4.4%, as previously published [4], to 6.1% (50/815).

The prevalence of *B*. *burgdorferi* s. l. during spring and autumn migration was 5.5% (11/201) and 6.4% (39/614), respectively (Tables 2 and 3). Both larvae (34%) and nymphs (66%) were infected. The genospecies of *B*. *burgdorferi* s. l. included *B*. *garinii* (60%), *B*. *valaisiana* (18%), *B*. *afzelii* (18%) and *B*. *burgdorferi* s. s. (2%). One sample could not be differentiated between *B*. *afzelii* or *B*. *spielmanii* due to low sequence quality and few differences in the sequenced region between the two *Borrelia* species. Ticks infected with *B*. *burgdorferi* s. l. were collected from eleven bird species (Table 4).

**Table 2:** Tick parasitisation of birds and prevalence of *Neoehrlichia mikurensis*, *Rickettsia helvetica*, *Anaplasma phagocytophilum* and *Borrelia burgdorferi* sensu lato in *Ixodes ricinus* collected from birds, spring 2008.

Bird species	No of birds parasitised/ no of birds ^a^	No. of nymphs examined	No. of larva examined	*N*. *mikurensis* (nymphs/larvae)	*R*. *helvetica* (nymphs/larvae)	*A*. *phagocytophilum* (nymphs/larvae)	*B*. *burgdorferi* s. l. (nymphs/larvae)
**Migrating birds**							
*Acrocephalus arundinaceus*	1/1	1		1 (1/-)			
*Acrocephalus palustris*	1/5	1					
*Acrocephalus scirpaceus*	1/3	1					
*Sylvia borin*	1/17	1					
*Sylvia communis*	3/28	4	1				
*Luscinia svecica*	1/1		1				
*Carduelis cannabina*	1/19	1					
*Carduelis cabaret*	1/1		1				
*Carpodacus erythrinus*	1/4	1					
*Coccothraustes coccothraustes*	1/1	2					
*Phylloscopus collybita* ^b^	1/65	1					
*Sylvia atricapilla* ^b^	1/43	1					
*Erithacus rubecula* ^b^	24/232	22	27		2 (2/-)		1 (1/-)
*Turdus iliacus* ^b^	1/4	7	1			3 (3/-)	2 (2/-)
*Turdus merula* ^b^	12/77	69	14	2 (2/-)	1 (1/-)	1 (1/-)	5 (5/-)
*Turdus pilaris* ^b^	3/29	4					1 (1/-)
*Turdus philomelos* ^b^	2/17	11	3				
*Fringilla coelebs* ^b^	1/14	1					
*Prunella modularis* ^b^	5/32	20	3	1 (1/-)			2 (2/-)
*Sturnus vulgaris* ^b^	1/16		1				
**Resident birds**							
*Carduelis carduelis*	1/5	1				1 (1/-)	
Total	**64/614**	**149**	**52**	**4 (4/-)**	**3 (3/-)**	**5 (5**/-)	**11 (11**/-)

^a^ The number of parasitised birds was previously published by Kjelland et al. (4).

^b^ Migratory birds, but some individuals may overwinter.

**Table 3:** Tick parasitisation of birds and prevalence of *Neoehrlichia mikurensis*, *Rickettsia helvetica*, *Anaplasma phagocytophilum* and *Borrelia burgdorferi* sensu lato in *Ixodes ricinus* collected from birds, autumn 2008.

Bird species	No. of birds parasitised/ no. of birds ^a^	No. of nymphs examined	No. of larvae examined	*N*. *mikurensis* (nymphs/larvae)	*R*. *helvetica* (nymphs/larvae)	*A*. *phagocytophilum* (nymphs/larvae)	*B*. *burgdorferi* s. l. (nymphs/larvae)	*B*. *miyamotoi* (nymphs/larvae)
**Migrating birds**	** **						** **	
*Acrocephalus scirpaceus*	1/5		1					
*Phylloscopus trochilus*	24/440	23	21			1 (1/-)	2 (2/-)	
*Sylvia curruca*	3/32	1	3					
*Sylvia communis*	17/93	18	31				3 (2/1)	
*Motacilla flava*	1/3	1						
*Oenanthe oenanthe*	1/99	1						
*Carduelis cabaret*	2/8	2	2					
*Carduelis cannabina*	1/41		1					
*Anthus trivialis*	7/19	10	8				1 (1/-)	
*Sylvia atricapilla* ^b^	6/135	5	10					
*Erithacus rubecula* ^b^	5/179	4	10					
*Turdus iliacus* ^b^	2/64	12	4				10 (7/3)	
*Turdus pilaris* ^b^	2/48	7						
*Turdus philomelos* ^b^	3/48	8	9				2 (-/2)	
*Turdus merula* ^b^	10/161	32	12	3 (3/-)		1 (1/-)	10 (7/3)	
*Emberiza schoeniclus* ^b^	2/26	2	1					
*Fringilla coelebs* ^b^	50/258	39	314	3 (2/1)			10 (3/7)	1 (-/1)
*Fringilla montifringilla* ^b^	1/48	1	2					
*Anthus pratensis* ^b^	2/59	1	1					
**Resident birds**								
*Cyanistes caeruleus*	4/1325	5	1					
*Lophophanes cristatus*	1/52	1						
*Parus major*	1/147		1					
*Carduelis chloris*	1/99		1					
*Troglodytes troglodytes*	4/150	1	5				1 (-/1)	
Not identified	1/1		2					
Total	**152/3539**	**174**	**440**	**6 (5/1)**	**0**	**2 (2**/-)	**39(22/17)**	**1 (-/1)**

^a^ The number of parasitised birds was previously published by Kjelland et al. (4).

^b^ Migratory birds, but some individuals may overwinter.

**Table 4:** *Borrelia* species in *Borrelia* infected *Ixodes ricinus* ticks.

Bird species	*Borrelia* infected ticks	*B*. *garinii* (nymphs/larvae)	*B*. *valaisiana* (nymphs/larvae)	*B*. *afzelii* (nymphs/larvae)	*B*. *burgdorferi* s. s. (nymphs/larvae)	*B*. *miyamotoi* (nymphs/larvae)	Undetermined (nymphs/larvae)
*Anthus trivialis*	1	1 (1/-)					
*Erithacus rubecula*	1			1 (1/-)			
*Fringilla coelebs*	11	9(2/7)		1 (1/-)		1 (-/1)	
*Phylloscopus trochilus*	2			1 (1/-)	1 (1/-)		
*Prunella modularis*	2	1 (1/-)		1 (1/-)			
*Sylvia communis*	3	1 (1/-)		1 (1/-)			1 (1/-)
*Troglodytes troglodytes*	1			1 (-/1)			
*Turdus iliacus*	12	12 (9/3)					
*Turdus merula*	15	3 (2/1)	9 (7/2)	3 (3/-)			
*Turdus philomelos*	2	2 (-/2)					
*Turdus pilaris*	1	1 (1/-)					
Total	**51**	**30 (17/13)**	**9 (7/2)**	**9 (8/1)**	**1 (1/-)**	**1 (-/1)**	**1 (1/-)**

In total, 1.2% (10/815) of the ticks were infected with *N*. *mikurensis*. The prevalence during spring and autumn migration was 2.0% (4/201) and 1.0% (6/614), respectively. One of the positive ticks was a larva co-infected with *B*. *garinii*. It was collected from a chaffinch (*Fringilla coelebs*). Nine other ticks were found on the same bird, but all were uninfected. In two cases, two positive nymphs were found on the same bird, a blackbird (*Turdus merula*) and a chaffinch (*F*. *coelebs*), respectively. Nymphs infected with *N*. *mikurensis* were also found on dunnock (*Prunella modularis*) and great reed warbler (*Acrocephalus arundinaceus*).

*Anaplasma phagocytophilum* was detected in 0.9% (7/815) of the ticks, whereof all were nymphs. The prevalence was 2.5% (5/201) and 0.3% (2/614) during spring and autumn migration, respectively. Three of the infected ticks collected during spring migration were collected from the same bird, a redwing (*T*. *iliacus*), which hosted a total of eight ticks. *A*. *phagocytophilum* infected ticks were also collected from goldfinch (*Carduelis carduelis*), blackbird (*T*. *merula*) and willow warbler (*Phylloscopus trochilus*).

The prevalence of *R*. *helvetica* was 0.4% (3/815). All three infected ticks were nymphs collected during spring migration. The ticks were collected from the bird species blackbird (*T*. *merula*) and European robin (*Erithacus rubecula*).

Although the *Borrelia* real-time PCR used targets the Lyme *Borreliae* (*B*. *burgdorferi* s. l.), one sample infected with a relapsing fever *Borrelia* (*B*. *miyamotoi*) was also detected. This tick larva was collected from a chaffinch (*F*. *coelebs*) during autumn migration. This bird had the highest tick infestation (n = 56) in the study. One other tick (a nymph) from this bird was infected with *B*. *afzelii*.

The prevalence of co-infection in infected ticks was 6.0% (4/67). These ticks were co-infected with either *B*. *garinii* and *N*. *mikurensis* (n = 2, 1 nymph, 1 larva), *B*. *garinii* and *A*. *phagocytophilum* (n = 1, nymph), or *B*. *afzelii* and *N*. *mikurensis* (n = 1, nymph).

## Discussion

Ticks collected from birds during spring and autumn migration in southern Norway were investigated for infection with *B*. *burgdorferi* s. l., *A*. *phagocytophilum*, *N*. *mikurensis* and *R*. *helvetica*. So far, only a few studies have investigated the tick-borne pathogens transported by avian hosts in this region, and the current study adds new information to this subject. Our study is the first to report *N*. *mikurensis*, *R*. *helvetica* and *B*. *miyamotoi* infection in ticks collected from birds in Norway.

The sample material has previously been investigated for *B*. *burgdorferi* s. l. and in the original study [4], DNA from the ticks was extracted using the phenol-chloroform method. However, a high percentage of PCR inhibition with this method has previously been reported [12]. For this reason, the samples were tested for inhibition, diluted and reanalysed for *B*. *burgdorferi* s. l. This enabled the detection of the spirochete in an additional 14 ticks, increasing the prevalence from 4.4%, as previously published [4], to 6.1%. Phenol and ethanol are PCR inhibitors if not completely removed during extraction [27]. This DNA extraction method is widely used [12, 28–30], and underestimation of prevalence of tick-borne pathogens in studies using this procedure is possible, unless methods to detect inhibition are applied. Further, engorged ticks contain blood, and blood also contains factors that may lead to inhibition of the PCR reaction [27]. Hence, quality control of the sample material to exclude inhibition is important for true prevalence estimations and reliable results.

The prevalence of *B*. *burgdorferi* s. l. detected in this study (6.1%) is lower than what is reported in ticks collected from birds from other European countries [3, 5, 6, 11]. A previous study investigating *B*. *burgdorferi* s. l. in ticks collected from migratory birds captured at the same site as in the present study reported a prevalence of 6.1% (8), which corresponds exactly with our results. The same study reported a higher prevalence at more easterly located bird observatories (13.3%) and concluded that this difference might be due to the low prevalence of *B*. *burgdorferi* s. l. in Great Britain [8, 31], whence many of the birds trapped at Lista Bird Observatory most likely migrate. The prevalence of *B*. *burgdorferi* s. l. in questing ticks in southern Norway ranges from 15% to 30% depending on study area [14, 32], and if the ticks were locally recruited by reservoir host birds for *B*. *burgdorferi* s. l. a higher prevalence might have been expected, although a borreliacidal effect of bird blood [33] cannot be excluded.

Of the *Borrelia* species investigated in this study, *B*. *garinii* was the most prevalent (60%) which is in accordance with other European studies [3, 6]. The blackbird and song thrush (both *Turdus* species) are known reservoir hosts for *B*. *garinii* [10, 11], and other bird species may have the same potential. In present study, larvae and nymphs infected with *B*. *garinii* were collected from eight bird species, whereof four were *Turdus* species. The other species were dunnock (*Prunella modularis*), chaffinch (*F*. *coelebs*), tree pipit (*Anthus trivialis*) and whitethroat (*Sylvia communis*). The infected larvae were collected from chaffinch (*F*. *coelebs*), in addition to *Turdus* species. Transovarial transmission of *B*. *burgdorferi* s. l. is rare or absent [34, 35], and the larvae are most likely infected through feeding, indicating that also chaffinch (*F*. *coelebs*) may contribute to the transmission of *B*. *garinii*. Of the pathogens investigated here, transovarial transmission has only been demonstrated for *R*. *helvetica* [36], and to a lesser extent *B*. *miyamotoi* [34], and for *A*. *phagocytophilum* only in the tick *Dermacentor albipictus* [37]. Apart from *R*. *helvetica*, the transmission rate for these pathogens is low, and the main route of infection is by feeding.

In the present study, the detected prevalence of *N*. *mikurensis* was 1.2%. In European countries, between 1.7% and 4.4% of ixodid ticks feeding on birds are infected with *N*. *mikurensis* [3, 5, 6, 16]. The prevalence in questing ticks in European countries, including Norway, ranges from <1 to >20%, depending on study area [12–14, 38]. However, *N*. *mikurensis* has not been detected in ticks in Great Britain [31, 39, 40], and migration from Great Britain may consequently lead to a low prevalence of the pathogen in ticks from birds trapped at Lista Bird Observatory during spring migration. However, in addition to blackbirds (*T*. *merula*), which mainly migrate to Norway from low prevalence areas in Great Britain, ticks infected with *N*. *mikurensis* were also collected from other bird species (e. g. *P*. *modularis*) which mainly migrate from mainland Europe [41] and a low prevalence of *N*. *mikurensis* infected ticks were seen also in these birds.

In the current study, *N*. *mikurensis* was detected in a larva collected from a common chaffinch (*F*. *coelebs*). *N*. *mikurensis*-infected larval *I*. *ricinus* feeding on Eurasian wren (*Troglodytes troglodytes*) and redwing (*T*. *iliacus*) have previously been reported by Heylen et al. [3]. Since transovarial transmission of *N*. *mikurensis* is assumed not to occur, infection is likely to have occurred during feeding [12, 16, 40]. Ticks cluster around the bird’s beak which may facilitate co-feeding [42]. If the larva did not acquire the bacterium from the bird, it might be explained by transmission via co-feeding, where an infected nymph may have transmitted the pathogen to the larvae and dropped off before tick-collection. However, evidence of birds facilitating co-feeding transmission or as reservoir hosts for *N*. *mikurensis* is lacking [17, 18], and more studies are needed to understand the importance of birds for the epidemiology of this pathogen. Furthermore, the low prevalence of *N*. *mikurensis* in ticks feeding on birds during autumn migration compared to the prevalence in questing ticks in the region, raise the question if passerine birds are incompetent reservoir hosts or not part of the transmission cycle of this pathogen. It has been suggested that *B*. *afzelii* are killed in *I*. *ricinus* that feed on pheasants [33], but whether similar mechanisms apply for *N*. *mikurensis* and passerine birds needs to be investigated.

Prevalence found in previous studies of *A*. *phagocytophilum* in ticks collected from birds in Europe ranges from 0 to 5% [3, 5, 6]. Our finding (0.9%) is within that range. Prevalence in questing ticks in Western Europe, including the UK, is typically below 10%, but prevalence >20% is also reported [19]. In southern Norway, prevalences between 1% and 2% have been found in questing ticks [14, 43]. In this study, *A*. *phagocytophilum* was detected in three ticks collected from a single redwing (*T*. *iliacus*), which may be a result of the host being infectious or, alternatively, a result of co-feeding. However, they were all nymphs and may have been infected during their previous blood meal. A few cases of birds infected with *A*. *phagocytophilum* have been reported [17, 44], which indicates the possibility of transmission to feeding ticks. However, the importance of birds for the infection cycle of *A*. *phagocytophilum* seems to be minor, although this is unclear and needs further investigation.

Only three ticks (0.4%) were infected with *R*. *helvetica*, all collected during spring migration. The three nymphs were collected from European robin (*E*. *rubecula*) and blackbird (*T*. *merula*), which mainly migrate to Norway from Great Britain and Western Europe [41]. The prevalence, 0.4%, is low compared to studies conducted in Europe, both in questing ticks [45, 46] and in ticks collected from birds [3, 17]. In Great Britain, a study reported a widespread distribution of *R*. *helvetica* in 4/116 questing *I*. *ricinus* [47]. Studies from southern Norway indicate that less than 2% of questing ticks are infected in the region where this current study was conducted (22, Kjelland, Myre, Pedersen, Kloster, & Jenkins, Submitted manuscript).

A larva collected from a chaffinch (*F*. *coelebs*) was infected with *B*. *miyamotoi*. This was a chance observation as the applied method is not optimized to detect this species; systematic investigations are thus likely to detect higher prevalences. *B*. *miyamotoi* has previously been found in spleen from a great tit (*P*. *major*) and a European greenfinch (*Carduelis chloris*) indicating systemic infection [48]. However, low prevalence (< 1%) is reported in ticks collected from different bird species [3, 6], demonstrating low transmission efficiency. Further studies are necessary to determine the importance of birds for the transmission cycle of this bacterium. *B*. *miyamotoi* is widespread in questing ticks in southern Norway, however a low prevalence (less than 1.5%) is reported [14, 49], and the reported prevalence in Europe is less than 4% [31, 45, 50, 51].

In humans, simultaneous infection with *B*. *burgdorferi* s. l. and other tick-borne pathogens are reported and this is suggested to make the disease more severe and/or prolonged [52, 53]. We observed ticks co-infected with *N*. *mikurensis* and *B*. *burgdorferi* s. l, and *A*. *phagocytophilum* and *B*. *burgdorferi* s. l. A previous study of questing ticks in Norway detected a high prevalence of co-infection with *N*. *mikurensis* and *B*. *afzelii* [14]. Correlation between *N*. *mikurensis* and *B*. *burgdorferi* s. l. has also been detected in ticks from birds [3]. We detected only three ticks with this combination and no association could be established. However, due to the small number of infections detected, such an association cannot be excluded.

Although all but one (a goldfinch, *Carduelis carduelis*) of the birds on which ticks were found were migratory species, it is not necessarily the case that the ticks were acquired outside Norway. Members of some migratory species remain in the country, while newly arrived birds may acquire ticks locally. The difference in pathogen prevalence between spring and autumn migration was not significant and, assuming the birds acquired their parasites along their migration route no difference in export and import of tick-borne pathogens is indicated. The prevalence of all the pathogens investigated here is lower than for the reported prevalence in questing ticks in Norway [13, 14, 32, 43]. These findings suggest that birds play a relatively minor part in the infectious cycle of tick-borne pathogens in Norway, although, since all pathogens investigated were found, they may be important in pathogen spread.

Only *I*. *ricinus* were found on birds in this present study, which is consistent with other results [5, 11]. This is the dominant tick species in Norway and Great Britain [54, 55], and the most common species found on passerine birds in Europe [3, 5–7, 9, 11, 16]. Another study investigating ticks on birds in Norway found 99% *I*. *ricinus* [56].

## Conclusion

The study demonstrates that all the known major tick-borne bacterial pathogens in southern Norway are subject to transport by migratory birds, potentially allowing introduction and establishment in new areas. This study is the first to report *N*. *mikurensis*, *R*. *helvetica* and *B*. *miyamotoi* in ticks collected from birds in Norway.

## Supporting information

S1 File (PDF)
