# Supplementary material for: Tick-borne pathogens in Ixodes ricinus ticks collected from migratory birds in southern Norway
Source: PLoS One. 2020 Apr 9;15(4):e0230579. doi: 10.1371/journal.pone.0230579 (PMC7145107; doi:10.1371/journal.pone.0230579)
Supplement: S1 File — (PDF) [file pone.0230579.s001.pdf]

# Manuscript Details

| | |
|---|---|
| **Manuscript number** | TTBDIS_2020_51 |
| **Title** | Widespread low-prevalence occurrence of Rickettsia helvetica in Ixodes ricinus ticks in southern Norway |
| **Article type** | Research Paper |

**Abstract**

Rickettsia helvetica is a tick-borne pathogen that may cause severe human disease. Knowledge of its distribution in Norway, where Ixodes ricinus reaches its northern limit, is very sparse. It was detected only recently in Norway, but it is prevalent and widely distributed in I. ricinus ticks in the neighboring countries Sweden and Denmark. In this study 2396 questing adult, nymphal and larval I. ricinus ticks were collected from two counties in Norway and analyzed for the presence of R. helvetica using a specific real-time PCR targeting the citrate synthase gene gltA. A further 495 nymphal I. ricinus from a third county was analyzed for Rickettsia spp. using a different method that is not species-specific. The overall prevalence was 1.6 %. Local variations were observed, but prevalence was < 5 % at all locations.

| | |
|---|---|
| **Keywords** | pyrosequencing; real-time PCR; sequencing; tick-borne pathogens |

| | |
|---|---|
| **Corresponding Author** | Vivian Kjelland |
| **Corresponding Author's Institution** | University of Agder & Sørlandet Hospital Health Enterprise |
| **Order of Authors** | Vivian Kjelland, Ingvild Bakken Myre, Benedikte Nevjen Pedersen, Hanne Kloster, Andrew Jenkins |

| | |
|---|---|
| **Suggested reviewers** | Peter Wilhemsson, snorre stuen |

## Submission Files Included in this PDF

**File Name [File Type]**

Cover letter_Rickettsia helvetica in Norway_Kjelland et al.docx [Cover Letter]

Rickettsia helvetica in Norway.docx [Manuscript File]

## Submission Files Not Included in this PDF

**File Name [File Type]**

Figure 1.png [Figure]

To view all the submission files, including those not included in the PDF, click on the manuscript title on your EVISE Homepage, then click 'Download zip file'.

Dear Editor(s),

We hope that our manuscript “Widespread low-prevalence occurrence of *Rickettsia helvetica* in *Ixodes ricinus* ticks in southern Norway” may be of value to the general readership of Ticks and Tick-Borne Diseases, and that it will be acceptable for publication in your journal.

The manuscript was initially submitted to Zoonoses and Public Health, however, was deemed of too limited public health significance for the journal (the full response can be seen below).

No other submissions/reports regarding this work has been done.

Sincerely, Vivian Kjelland & co-authors

<u>Full response from Zoonoses and Public Health:</u>
Dear Dr. Kjelland:

I write you in regards to manuscript # ZPH-Jan-20-012.R1 entitled "Widespread low-prevalence occurrence of Rickettsia helvetica in Ixodes ricinus ticks in southern Norway" which you submitted to Zoonoses and Public Health.

In view of the criticisms of the reviewer(s) found at the bottom of this letter, your manuscript has been denied publication in the Zoonoses and Public Health.

Thank you for considering the Zoonoses and Public Health for the publication of your research. I hope the outcome of this specific submission will not discourage you from the submission of future manuscripts.

Kind regards,
Dr. Jonathan Oliver
Associate Editor, Zoonoses and Public Health joliver@umn.edu

Reviewer(s)' Comments to Author:
The manuscript is of too limited public health significance. I recommend that you submit it elsewhere.

**Title: Widespread low-prevalence occurrence of *Rickettsia helvetica* in *Ixodes ricinus* ticks in southern Norway**

**Running title: *Rickettsia helvetica* in Norway**

Vivian Kjelland[a,b,*], Ingvild Bakken Myre[a], Benedikte Nevjen Pedersen[c], Hanne Kloster[a], Andrew Jenkins[c]

[a]University of Agder, Faculty of Engineering and Science, Department of Natural Sciences, Gimlemoen 25, NO-4630 Kristiansand, Norway

[b]Sørlandet Hospital Health Enterprise, Research Unit, P. O. Box 416, NO-4604 Kristiansand, Norway

[c]University of South-Eastern Norway, Department of Natural Sciences and Environmental Health, Gullbringveien 38, NO-3800 Boe, Norway

[*]Corresponding author at: Department of Natural Sciences, University of Agder, Gimlemoen 25, 4630 Kristiansand, Norway. Tel.: +47 38 14 10 00. E-mail address: vivian.kjelland@uia.no (V. Kjelland).

**Highlights**

- First description of the prevalence and distribution of *R. helvetica* in *I. ricinus* ticks in Norway
- The pathogen was detected in all the three investigated counties
- Increased *I. ricinus* distribution & abundance lead to increased need for awareness

## Abstract

*Rickettsia helvetica* is a tick-borne pathogen that may cause severe human disease. Knowledge of its distribution in Norway, where *Ixodes ricinus* reaches its northern limit, is very sparse. It was detected only recently in Norway, but it is prevalent and widely distributed in *I. ricinus* ticks in the neighboring countries Sweden and Denmark. In this study 2396 questing adult, nymphal and larval *I. ricinus* ticks were collected from two counties in Norway and analyzed for the presence of *R. helvetica* using a specific real-time PCR targeting the citrate synthase gene *gltA*. A further 495 nymphal *I. ricinus* from a third county was analyzed for *Rickettsia* spp. using a different method that is not species-specific. The overall prevalence was 1.6 %. Local variations were observed, but prevalence was < 5 % at all locations.

**Keywords:** pyrosequencing; real-time PCR; sequencing; tick-borne pathogens

## Introduction

*Rickettsia helvetica* is an emerging tick-borne pathogen mainly transmitted by *Ixodes ricinus* (Portillo et al., 2015). The bacterium was detected as early as 1979 in Switzerland, and it was confirmed to be a new member of the spotted fever group *Rickettsiae* (SFGR) in 1993 (Beati et al., 1993). Since its discovery, *R. helvetica* has been detected in many parts of Europe, including Scandinavia (Nilsson et al., 1997, Nielsen et al., 2004, Oteo and Portillo, 2012), and in other parts of the world, including Russia, South Africa and Thailand (Aung et al., 2014, Kartashov et al., 2017, Essbauer et al., 2018). Despite its widespread distribution, relatively few human cases have been reported. *R. helvetica* infections are primarily considered mild and self-limiting with un-specific symptoms such as fever, headache, myalgia or rash (Oteo and Portillo, 2012, Lindblom et al., 2016). However, the bacteria have also been isolated from cerebrospinal fluid of patients with meningitis of uncertain aetiology, and two cases of sudden cardiac death with perimyocarditis related to *R. helvetica* infection were reported in Sweden in 1999 (Nilsson et al., 1999, Nilsson et al., 2010).

The reported infection prevalence in host-seeking *I. ricinus* in Europe is typically below 20 %, but varies from 1 % to 66 % (Sprong et al., 2009, Oteo and Portillo, 2012). The reasons for these striking differences in prevalence in various locations is still unknown, but may be due to a combination of biotic factors such as reservoir capacity of the local tick host animals, and abiotic factors such as local climatic conditions, and further studies are necessary to elucidate the ecological cycle of the pathogen. *R. helvetica* was recently detected in Norway, though a very low prevalence was reported: 2/600 (0.3 %) adult *I. ricinus* ticks was infected (Quarsten et al., 2015). However, further studies are necessary to describe the true infection prevalence in ticks in Norway. In the present study we investigated the prevalence of *R. helvetica* in 2891 *I. ricinus* ticks collected from 14 sites in southern Norway.

## Materials and methods

**Tick collection and DNA extraction**

Host-seeking *I. ricinus* ticks were collected from 14 sites in the Norwegian counties Vestfold og Telemark, Agder and Vestland (Table 1, Figure 1). The ticks were collected by flagging the undergrowth as previously described (Kjelland et al., 2010).  The ticks were placed in plastic tubes containing 70 % ethanol and kept at 4°C until DNA extraction. Only *I. ricinus* ticks were found.

DNA was extracted by the commercial kit DNeasy® Blood & Tissue Kit (Qiagen, Germany) with some modifications as previously described (Kjelland et al., 2010) *or* by phenol-chloroform extraction (Halos et al., 2004) *or* by digestion with ammonium hydroxide (Jenkins et al., 2019) (Table 1). The 495 ticks from Vestfold og Telemark were pooled in groups with 5 ticks in each pool after extraction of DNA. Purified DNA was stored at -20°C until further analysis.

**_Rickettsia helvetica_ specific real-time PCR**

DNA extracts from the ticks collected in the counties Agder and Vestland were examined for *R. helvetica* by using a real-time PCR assay with primers and probe specific for a region of the *gltA* gene (Table 2). Real-time PCR was performed using StepOnePlus Real Time PCR System (Applied Biosystems Inc. (ABI), California, USA). The PCR mixture contained 10 µl TaqMan© Environmental DNA Master Mix 2.0 (ABI), 800 nM of each *Rh* primer (ABI), 800 nM *Rh* probe (ABI), 5 µl of template DNA and dd$H_2O$ to the total reaction volume of 20 µl. The PCR conditions were as follows: 40°C for 2 min and 95°C for 10 min, followed by 47 cycles of 95°C for 15 s, 56°C for 30 s and 72°C for 20 s. Optical detection of fluorescence intensity was done after each cycle. A synthetic plasmid containing the *glt*A sequence (GenBank accession number KU310588; the length of the *glt*A gene is 1308 bp, and the 101 bp real-time PCR target sequence corresponds to positions 907-1007) cloned into the vector pUC57 was constructed according to our specifications and obtained from GenScript (New Jersey, USA) and used as a positive control. Positive and negative controls were included in all runs.

**_Rickettsia_ spp. real-time PCR**

The pooled DNA samples from Vestfold og Telemark county were examined for *Rickettsia spp.* as described by Stenos et al. (2005) with minor modifications. Briefly, the PCR mixture included 10 µl TaqMan® Universal PCR Master Mix (ABI), 800 nM of each *R*spp. primer (Eurofins Genomics, Ebersberg, Germany), 800 nM *R*spp. probe (ABI), 5 µl of template DNA and ddH2O to the total reaction volume of 20 µl. The PCR conditions were as follows: 40°C for 2 min and 95°C for 10 min, followed by 47 cycles of 95°C for 15 s, 58°C for 30 s and 72°C for 20 s. Due to lack of material, the samples were not analyzed by the species specific real-time PCR or sequencing. *Rickettsia conorii* (Vircell, Granada, Spain) was used as positive control. Positive and negative controls were included in all runs.

**Direct sequencing and pyrosequencing**

Real-time PCR positive samples were subjected to direct sequencing or pyrosequencing of *gltA*. Briefly, in the direct sequencing the real-time PCR positive samples were re-amplified with a standard PCR where every reaction consisted of 2.5 µl 10XPCR Gold Buffer (ABI), 2.5 µl dNTP Mix (ABI), 2.5 µl $MgCl_2$ Solution (ABI), 0.2 µl AmpliTaq Gold® (ABI), 800 nM of each *Rh* primer, 5 µl template DNA and $ddH_2O$ to the total reaction volume of 25 µl. The cycling parameters were: 40°C for 2 min, 95°C for 10 min, then 47 cycles of 95°C for 15 s, 56°C for 30 s and 72°C for 20 s. PCR products were purified with ExoSAP-IT® (Affymetrix, California, USA) following the manufacturer's instructions, sequenced in both directions using BigDye® Terminator v.1.1 Cycle sequencing RR-100 (ABI), and analyzed with a 3130 Genetic Analyzer automated capillary sequencer (ABI).

Before pyrosequencing the real-time PCR positive samples were re-amplified on the RotorGene Q (Qiagen GmbH, Hilden, Germany) using a biotinylated forward primer to achieve streptavidin binding in the pyrosequencing preparation. The reaction mixture contained 10 µl TaqMan® Universal Master Mix II, with UNG (ABI), 250 nM biotin labelled *Rh* forward primer, 250 nM *Rh* revers primer, 300 nM *Rh* probe, 5 µl template DNA and RNase free water to the total reaction volume of 20 µl. The cycling parameters were: 50°C for 2 min, 95°C for 10 min, then 48 cycles of 95°C for 15 s, 56°C for 30 s and 72°C for 20 s.  The real-time PCR products were analyzed on the Pyromark Q24 (Qiagen) according to the manufacturer's instructions, using 300 nM *Rh* revers primer and Pyrogold SQA reagents (Qiagen). Negative and positive controls were included in each run. The pyrogram of each sample was compared with the pyrogram of the positive control to determine true positive samples.

## Results

In total, 2891 *I. ricinus* ticks were analyzed; 2165 from Agder county, 495 from Vestfold og Telemark county and 231 from Vestland county. Ticks collected in Vestfold og Telemark county was analyzed

for the presence of *Rickettsia* spp., whereas the ticks collected in the two remaining counties were analyzed with a real-time PCR specific for *R. helvetica* (Table 3).

A total of 45/2396 samples yielded a positive *R. helvetica* real-time PCR result (Table 3). All 45 PCR products yielded the expected fragment length (101 bp) when analyzed by agarose gel electrophoresis. Of these, 11/45 samples were verified by direct sequencing or pyrosequencing. In Vestfold og Telemark, *Rickettsia* spp. was detected in one tick pool.

## Discussion

The present study is the first to describe the prevalence of *R. helvetica* in host-seeking *I. ricinus* ticks in Norway. In accordance with other European studies (Oteo and Portillo, 2012), a widespread distribution of *R. helvetica* was found in southern Norway.

The pathogen was detected in all three investigated counties, indicating a widespread distribution in Norway. The infection prevalence was low throughout the sampling region, 3.9 %, 1.7 % and 0.2 % in ticks collected in Vestland, Agder and Vestfold og Telemark, respectively.

All samples positive in the *R. helvetica* specific real-time PCR were further analyzed in a direct sequencing or a pyrosequencing assay. Eleven of 45 samples were successfully sequenced, and only *R. helvetica* was detected. As several samples are close to the detection limit of the assays, it is not possible to conclusively determine whether the samples that were not successfully sequenced are false positives in real-time PCR, or true positives below the detection limit of the sequencing assays, although estimation of the real-time PCR sensitivity and specificity favors the latter interpretation (data not shown). Furthermore, agarose gel electrophoresis analysis of all samples positive in the *R. helvetica* specific real-time PCR yielded amplicons of the expected size (101 base pairs). Thus, although there is a degree of uncertainty regarding the exact prevalence of the pathogen, the study

demonstrates a low infection rate of the pathogen in southern Norway. Correspondingly, the pathogen was previously detected in 0.3 % *I. ricinus* ticks collected in Agder county (Quarsten et al., 2015), which is consistent with the low prevalence found in this study.

Most samples were analyzed using a real-time PCR specific for *R. helvetica*, however one set of samples was analyzed using a previously-published generic primer set for *Rickettsia* spp. One positive pool was detected, giving a minimum infection rate at 0.2 %. Unfortunately, genotyping of this sample could not be done due to lack of material. However, since most previous studies conducted in the Nordic countries have shown *R. helvetica* to be the predominant *Rickettsia* species in *I. ricinus* ticks in Scandinavia (Table 4), it may be assumed, although not conclusively, that the detected *Rickettsia* spp. was in fact *R. helvetica*.

Interestingly, in the countries neighboring Norway the prevalence is significantly higher; in Sweden, the prevalence of *R. helvetica* in *I. ricinus* ticks reaches 17 % (Nilsson et al., 1999, Severinsson et al., 2010), whereas in Denmark up to 14 % is reported (Nielsen et al., 2004, Svendsen et al., 2009, Kantso et al., 2010, Michelet et al., 2014) (Table 4). This may reflect differences in methodology, although the methods used in present study coincided in part with those in the other studies (Table 4), in which case the results should be comparable. While it cannot be conclusively excluded that the observed differences between Norway and neighboring countries may be due to methodological differences it is well-known that the prevalence of pathogens in ticks varies between geographical regions, and seems to be influenced by the nature of the habitat, in particular which tick hosts that are found in the area.

## Conclusions

Due to the substantial difference in the prevalence of *R. helvetica* in Norway and the neighboring countries, Sweden and Denmark, it is important to conduct further research in Norway to determine

if the prevalence is in fact significantly lower in this region, and if so to investigate the reasons for this discrepancy. The occurrence and pathogenicity of *R. helvetica* in humans in Norway remains largely unaddressed, probably due to nonspecific symptoms, unawareness and the lack of diagnostic tools. Although SFGR other than *R. helvetica* are rarely reported in Scandinavia, future studies should aim to determine whether other species are present or absent in Norway. Recently, studies have indicated an increased distribution and abundance of *I. ricinus* ticks in Norway (Hvidsten et al., 2015, Kjaer et al., 2019), which may lead to an increase in the number of human and animal tick-borne disease, and awareness of new emerging pathogens and their infections is increasingly important.

## CRediT author statement

**Vivian Kjelland**: Conceptualization, Methodology, Investigation, Validation, Writing- Original draft preparation, Visualization, Supervision, Project administration, Funding acquisition. **Ingvild Myre Bakken**: Methodology, Investigation, Validation, Writing- Review & Editing. **Benedikte Nevjen Pedersen**: Methodology, Investigation, Validation, Writing- Review & Editing. **Hanne Kloster**: Methodology, Investigation, Validation, Writing- Review & Editing. **Andrew Jenkins**: Conceptualization, Methodology, Investigation, Validation, Writing- Review & Editing, Supervision.

## Declaration of Competing Interest

The authors declare no competing interests.


## Acknowledgements

This work was supported by the Interreg V Program (the ScandTick Innovation project, grant no. 20200422). We are grateful to Åshild Andreassen at the Norwegian Institute of Public Health for providing reagents and letting us use their laboratory facilities for the pyrosequencing assay. We are also grateful to Kristine Jensen, Martine Mesel, Silje Bersagel Skjæveland, Katrine Alice Broen and

Anna Grytaas for performing parts of the tick collection, DNA extraction and/or real-time PCR analyses.

## References

Aung, A. K., D. W. Spelman, R. J. Murray and S. Graves (2014). Rickettsial infections in Southeast Asia: implications for local populace and febrile returned travelers. Am J Trop Med Hyg **91**(3): 451-460. doi:10.4269/ajtmh.14-0191

Beati, L., O. Peter, W. Burgdorfer, A. Aeschlimann and D. Raoult (1993). Confirmation that Rickettsia helvetica sp. nov. is a distinct species of the spotted fever group of rickettsiae. Int J Syst Bacteriol **43**(3): 521-526. DOI:10.1099/00207713-43-3-521

Essbauer, S., M. Hofmann, C. Kleinemeier, S. Wolfel and S. Matthee (2018). Rickettsia diversity in southern Africa: A small mammal perspective. Ticks Tick Borne Dis **9**(2): 288-301. DOI:10.1016/j.ttbdis.2017.11.002

Halos, L., T. Jamal, L. Vial, R. Maillard, A. Suau, A. Le Menach, H. J. Boulouis and M. Vayssier-Taussat (2004). Determination of an efficient and reliable method for DNA extraction from ticks. Vet Res **35**(6): 709-713. DOI:10.1051/vetres:2004038

Hvidsten, D., F. Stordal, M. Lager, B. Rognerud, B. E. Kristiansen, A. Matussek, J. Gray and S. Stuen (2015). Borrelia burgdorferi sensu lato-infected Ixodes ricinus collected from vegetation near the Arctic Circle. Ticks Tick Borne Dis **6**(6): 768-773. DOI:10.1016/j.ttbdis.2015.07.002

Jenkins, A., C. Raasok, B. N. Pedersen, K. Jensen, A. Andreassen, A. Soleng, K. S. Edgar, H. H. Lindstedt, V. Kjelland, S. Stuen, D. Hvidsten and B. E. Kristiansen (2019). Detection of Candidatus Neoehrlichia mikurensis in Norway up to the northern limit of Ixodes ricinus distribution using a novel real time PCR test targeting the groEL gene. BMC Microbiol **19**(1): 199. DOI:10.1186/s12866-019-1502-y

Kantso, B., C. B. Svendsen, P. M. Jensen, J. Vennestrom and K. A. Krogfelt (2010). Seasonal and habitat variation in the prevalence of Rickettsia helvetica in Ixodes ricinus ticks from Denmark. Ticks Tick Borne Dis **1**(2): 101-103. DOI:10.1016/j.ttbdis.2010.01.004

Kartashov, M. Y., L. I. Glushkova, T. P. Mikryukova, I. V. Korabelnikov, Y. I. Egorova, N. L. Tupota, E. V. Protopopova, S. N. Konovalova, V. A. Ternovoi and V. B. Loktev (2017). Detection of Rickettsia helvetica and Candidatus R. tarasevichiae DNA in Ixodes persulcatus ticks collected in Northeastern European Russia (Komi Republic). Ticks Tick Borne Dis **8**(4): 588-592. DOI:10.1016/j.ttbdis.2017.04.001

Kjaer, L. J., A. Soleng, K. S. Edgar, H. E. H. Lindstedt, K. M. Paulsen, A. K. Andreassen, L. Korslund, V. Kjelland, A. Slettan, S. Stuen, P. Kjellander, M. Christensson, M. Teravainen, A. Baum, K. Klitgaard and R. Bodker (2019). Predicting and mapping human risk of exposure to Ixodes ricinus nymphs using

climatic and environmental data, Denmark, Norway and Sweden, 2016. Euro Surveill **24**(9). DOI:10.2807/1560-7917.ES.2019.24.9.1800101

Kjelland, V., S. Stuen, T. Skarpaas and A. Slettan (2010). Prevalence and genotypes of Borrelia burgdorferi sensu lato infection in Ixodes ricinus ticks in southern Norway. Scandinavian Journal of Infectious Diseases **42**(8): 579-585. DOI:10.3109/00365541003716526

Lindblom, A., K. Wallmenius, J. Sjowall, L. Fryland, P. Wilhelmsson, P. E. Lindgren, P. Forsberg and K. Nilsson (2016). Prevalence of Rickettsia spp. in Ticks and Serological and Clinical Outcomes in Tick-Bitten Individuals in Sweden and on the Aland Islands. PLoS One **11**(11): e0166653. DOI:10.1371/journal.pone.0166653

Michelet, L., S. Delannoy, E. Devillers, G. Umhang, A. Aspan, M. Juremalm, J. Chirico, F. J. van der Wal, H. Sprong, T. P. Boye Pihl, K. Klitgaard, R. Bodker, P. Fach and S. Moutailler (2014). High-throughput screening of tick-borne pathogens in Europe. Front Cell Infect Microbiol **4**: 103. DOI:10.3389/fcimb.2014.00103

Nielsen, H., P. E. Fournier, I. S. Pedersen, H. Krarup, T. Ejlertsen and D. Raoult (2004). Serological and molecular evidence of Rickettsia helvetica in Denmark. Scand J Infect Dis **36**(8): 559-563. DOI:10.1080/00365540410020776

Nilsson, K., K. Elfving and C. Pahlson (2010). Rickettsia helvetica in patient with meningitis, Sweden, 2006. Emerg Infect Dis **16**(3): 490-492. DOI:10.3201/eid1603.090184

Nilsson, K., T. G. Jaenson, I. Uhnoo, O. Lindquist, B. Pettersson, M. Uhlen, G. Friman and C. Pahlson (1997). Characterization of a spotted fever group Rickettsia from Ixodes ricinus ticks in Sweden. J Clin Microbiol **35**(1): 243-247.

Nilsson, K., O. Lindquist, A. J. Liu, T. G. Jaenson, G. Friman and C. Pahlson (1999). Rickettsia helvetica in Ixodes ricinus ticks in Sweden. J Clin Microbiol **37**(2): 400-403.

Nilsson, K., O. Lindquist and C. Pahlson (1999). Association of Rickettsia helvetica with chronic perimyocarditis in sudden cardiac death. Lancet **354**(9185): 1169-1173. DOI:10.1016/S0140-6736(99)04093-3

Oteo, J. A. and A. Portillo (2012). Tick-borne rickettsioses in Europe. Ticks Tick Borne Dis **3**(5-6): 271-278. DOI:10.1016/j.ttbdis.2012.10.035

Portillo, A., S. Santibanez, L. Garcia-Alvarez, A. M. Palomar and J. A. Oteo (2015). Rickettsioses in Europe. Microbes Infect **17**(11-12): 834-838. DOI:10.1016/j.micinf.2015.09.009

Quarsten, H., T. Skarpaas, L. Fajs, S. Noraas and V. Kjelland (2015). Tick-borne bacteria in Ixodes ricinus collected in southern Norway evaluated by a commercial kit and established real-time PCR protocols. Ticks and Tick-borne Diseases **6**(4): 538-544. DOI:10.1016/j.ttbdis.2015.04.008

Severinsson, K., T. G. Jaenson, J. Pettersson, K. Falk and K. Nilsson (2010). Detection and prevalence of Anaplasma phagocytophilum and Rickettsia helvetica in Ixodes ricinus ticks in seven study areas in Sweden. Parasit Vectors **3**: 66. DOI:10.1186/1756-3305-3-66

Skarphedinsson, S., B. F. Lyholm, M. Ljungberg, P. Sogaard, H. J. Kolmos and L. P. Nielsen (2007). Detection and identification of Anaplasma phagocytophilum, Borrelia burgdorferi, and Rickettsia helvetica in Danish Ixodes ricinus ticks. APMIS **115**(3): 225-230. DOI:10.1111/j.1600-0463.2007.apm_256.x

Sormunen, J. J., R. Penttinen, T. Klemola, J. Hanninen, I. Vuorinen, M. Laaksonen, I. E. Saaksjarvi, K. Ruohomaki and E. J. Vesterinen (2016). Tick-borne bacterial pathogens in southwestern Finland. Parasit Vectors **9**: 168. DOI:10.1186/s13071-016-1449-x

Sprong, H., P. R. Wielinga, M. Fonville, C. Reusken, A. H. Brandenburg, F. Borgsteede, C. Gaasenbeek and J. W. van der Giessen (2009). Ixodes ricinus ticks are reservoir hosts for Rickettsia helvetica and potentially carry flea-borne Rickettsia species. Parasit Vectors **2**(1): 41. DOI:10.1186/1756-3305-2-41

Stenos, J., S. R. Graves and N. B. Unsworth (2005). A highly sensitive and specific real-time PCR assay for the detection of spotted fever and typhus group Rickettsiae. Am J Trop Med Hyg **73**(6): 1083-1085. DOI:10.4269/ajtmh.2005.73.1083

Svendsen, C. B., K. A. Krogfelt and P. M. Jensen (2009). Detection of Rickettsia spp. in Danish ticks (Acari: Ixodes ricinus) using real-time PCR. Scand J Infect Dis **41**(1): 70-72. DOI:10.1080/00365540802530653

Wallmenius, K., J. H. Pettersson, T. G. Jaenson and K. Nilsson (2012). Prevalence of Rickettsia spp., Anaplasma phagocytophilum, and Coxiella burnetii in adult Ixodes ricinus ticks from 29 study areas in central and southern Sweden. Ticks Tick Borne Dis **3**(2): 100-106. DOI:10.1016/j.ttbdis.2011.11.003

**Table 1.** Overview of tick collection sites, time of sampling, the instar distribution, and the method used for DNA extraction.

| County | Site no. | Year/month of sampling | Number of ticks collected (adults/nymphs/larvae/instar not determined) | DNA extraction method† |
|---|---|---|---|---|
| Vestfold og Telemark | 1 | 2012/9 | 495 (0/495/0/0) | A |
| Agder | 2 | 2012/5; 2012/8; 2013/5; 2013/8; 2014/5; 2014/8 | 1267 (112/528/627/0) | P |
| | 3 | 2017/10 | 24 (18/6/0/0) | P |
| | 4 | 2016/8 | 4 (4/0/0/0) | Q |
| | 5 | 2016/8 | 13 (5/7/1/0) | Q |
| | 6 | 2016/8 | 7 (4/3/0/0) | Q |
| | 7 | 2016/8 | 187 (174/13/0/0) | Q |
| | 8 | 2016/8 | 24 (15/9/0/0) | Q |
| | 9 | 2017/8; 2017/9 | 109 (58/51/0/0) | P |
| | 10 | 2017/8 | 60 (0/0/0/60) | P |
| | 11 | 2017/7; 2017/8 | 122 (49/71/2/0) | P |
| | 12 | 2017/10 | 112 (31/46/0/35) | P |
| | 13 | 2016/9 | 236 (36/131/69/0) | Q |
| Vestland | 14 | 2017/10 | 231 (8/164/0/59) | P |
| **Total** | | | **2891 (514/1524/699/154)** | |

†DNA extraction method: A = digestion by ammonium hydroxide; P = phenol chloroform extraction; Q = DNeasy blood and tissue kit, Qiagen.

**Table 2.** Sequences for primers and probes used in this study.

| | Sequence (5’ – 3’) | Reference |
|---|---|---|
| *Rh* forward primer† | 5’-CCGTTTAGGTTAATAGGCTTCGG | This study |
| *Rh* reverse primer† | 5’-CCGAGTTCCTTTAATACTTCCTTACA | |
| *Rh* probe† | 5’-6-FAM-CGATCCACGTGCCGCAGTACT-MGBNFQ | |
| *R*spp. forward primer‡ | 5’-TCGCAAATGTTCACGGTACTTT | Stenos et al., 2005 |
| *R*spp. reverse primer‡ | 5’-TCGTGCATTTCTTTCCATTGTG | |
| *R*spp. probe‡ | 5’-6-FAM-TGCAATAGCAAGAACCGTAGGCTGGATG-BHQ-1 | |

† PCR target: part of the *gltA* gene specific for *Rickettsia helvetica*

‡PCR target: part of the *gltA* gene detecting *Rickettsia* spp.

**Table 3.** *Rickettsia helvetica* and *Rickettsia* spp. detected by real-time PCR in questing *Ixodes ricinus* ticks collected in three counties in Southern Norway.

| County | Total, % (n/N) | Adults, % (n/N) | Nymphs, % (n/N) | Larvae, % (n/N) | Instar not determined (n/N) |
|---|---|---|---|---|---|
| Vestfold og Telemark (*R*spp†) | 0.2 (1/495) ‡ | | 1/495† | | 0 |
| Agder (*Rh*†) | 1.7 (36/2165) | 13/506 | 19/865 | 2/699 | 2/95 |
| Vestland (*Rh*†) | 3.9 (9/231) | 0/8 | 7/164 | | 2/59 |
| Total | 1.6 (46/2891) | 2.5 (7/514) | 1.8 (26/1524) | 0.3 (2/699) | 1/154 |

†*R*spp: *Rickettsia* spp.; *Rh*: *Rickettsia helvetica*.

‡Ticks collected in Vestfold og Telemark was analyzed in pools of 5. Assuming only one infected individual in the pool, this yields a minimum infection rate of 0.2 %.

**Table 4.** Prevalence of *Rickettsia helvetica* in *Ixodes ricinus* ticks collected in the Nordic countries (Norway, Sweden, Denmark, Finland and Iceland). To our knowledge, the pathogen is not detected in Iceland.

| Country | DNA extraction method† | PCR target | *Rickettsia* species other than *R. helvetica* | *R. helvetica* prevalence |
|---|---|---|---|---|
| Norway | CK *or* P *or* A | *Rickettsia* spp. or *R. helvetica* | Not analyzed | 1.6 % (present study) |
| | CK | *Rickettsia* spp. | No | 0.3 % (Quarsten, Skarpaas et al. 2015) |
| Sweden | P | *Rickettsia* spp. | No | 1.2-11.2 % (Lindblom, Wallmenius et al. 2016) |
| | P | *Rickettsia* spp. | 1/887: possible *Rickettsia sibirica* | 9.5-9.6 % (Wallmenius, Pettersson et al. 2012) |
| | P | *Rickettsia* spp. | No | 1.5-17.3 % (Severinsson, Jaenson et al. 2010) |
| | P | *Rickettsia* spp. | No | MIR 16 %‡ (Nilsson, Lindquist et al. 1999) |
| | P | *Rickettsia* spp. | No | 1.7 % (Nilsson, Jaenson et al. 1997) |
| Denmark | A | *Rickettsia* spp. | No | 4.7 % (Kantso, Svendsen et al. 2010) |
| | A | *Rickettsia* spp. | No | 13 % (Svendsen, Krogfelt et al. 2009) |
| | CK *or* A | *R. helvetica* | Not analyzed | 0.1 % (Skarphedinsson, Lyholm et al. 2007) |
| | P | *Rickettsia* spp. | No | 4 % (Nielsen, Fournier et al. 2004) |
| | CK | *Rickettsia spp.* | No | 14 % (Michelet, Delannoy et al. 2014) |
| Finland | CK | *Rickettsia* spp. | 1/3169: *R. monacensis* | 1.1-5.1 % (Sormunen, Penttinen et al. 2016) |

†DNA extraction method: A = digestion by ammonium hydroxide; P = phenol chloroform extraction; CK = Commercial kit (DNeasy blood and tissue kit, Qiagen *or* The Qiagen DNA Mini kit, Qiagen *or* QIAampA DNA mini kits, Qiagen *or* NucleoSpin® RNA/DNA buffer sets, Macherey-Nagel *or* Wizard genomic DNA purification kit, Promega).

‡The authors estimated the average minimum and maximum infection prevalence to be 16 % and 36,8 %, respectively.

**Legend to Figure 1**

Host-seeking Ixodes ricinus ticks were collected from 14 sites in 3 counties in southern Norway; Jomfruland (1) in Vestfold og Telemark county, Tromøy (2), Tveit (3), Rogeheia (4), Eikelandsdalen (5), Bommen (6), Ravnås (7), Kvarstein (8), Eg (9), Baneheia (10), Odderøya (11), Voiebyen (12) and Trysnes (13) in Agder county, and Osterøy (14) in Vestland county (map created in QGIS 3.10.1).
